# Huge gastric bezoar caused by honeycomb, an unusual complication of health faddism: a case report

**DOI:** 10.1186/1757-1626-2-7077

**Published:** 2009-05-15

**Authors:** Panagiotis Katsinelos, Ioannis Pilpilidis, Grigoris Chatzimavroudis, Taxiarchis Katsinelos, Georgia Lazaraki, Kostas Fasoulas, Christos Zavos, Jannis Kountouras

**Affiliations:** 1 Department of Endoscopy and Motility Unit“G.Gennimatas” General Hospital, Ethnikis Aminis 41, Thessaloniki, 54635Greece; 2 Department of Gastroenterology, Second Department of Internal MedicineIppokration Hospital, Aristotle University of Thessaloniki, Konstantinoupoleos 49, Thessaloniki, 54624Greece

## Abstract

We report a young healthy woman, who believed that the consumption of large amounts of honeycomb would lead to good health and who finally developed a huge gastric bezoar of hard consistency. The conventional endoscopic techniques failed to manage the bezoar. Using the combination of injection of hydrogen peroxide 3% solution inside the bezoar to induce disintegration and a special designed needle-knife sphincterotome (bezotome) we managed to remove the bezoar in fragments. To the best of our knowledge this is the first reported bezoar caused by honeycomb.

## Introduction

Bezoars are foreign bodies found mainly in the stomach, which are composed of plant and vegetables (phytobezoars), persimmous (diospyrobezoars), hair (trichobezoars), milk (lactobezoars) or other bezoars [1]. Their management includes a wide spectrum of treatment options, from conservative treatment to surgery or endoscopic intervention [1,2].

We describe the first case of a huge bezoar of very hard consistency, made from honeycomb, which required sophisticated endoscopic techniques for its removal.

## Case Presentation

A 44-year-old Greek woman was referred to our department for endoscopic treatment of a huge gastric bezoar. Past medical history of the patient revealed daily consumption of large quantities of honeycomb during the last 2 months, because she believed that the honeycomb might have beneficial effect on the irritable bowel syndrome and on her health in general. Physical examination and laboratory data were unremarkable. During last ten days, she presented episodes of epigastric pain associated with nausea, especially after eating. Despite the initiation of treatment with proton pump inhibitors, the symptoms were not relieved. Upper endoscopy performed by a private gastroenterologist disclosed a yellow coloured huge bezoar, very hard to touch with forceps.

**Figure 1:**
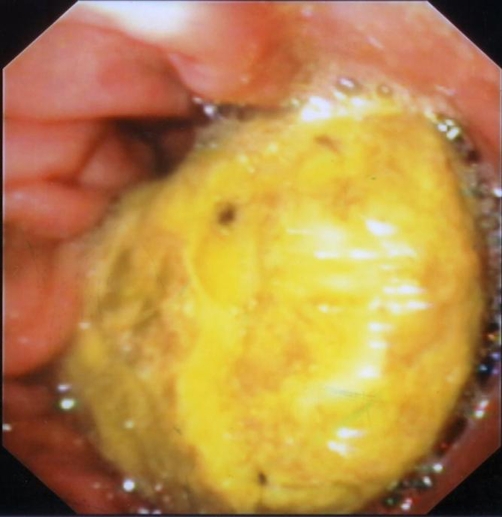
A huge honeycomb-bezoar occupying the gastric lumen.

We performed the intervention with propofol administration to achieve a well-sedated patient. Endoscopic examination of stomach confirmed previous findings (Figure 1). We tried to fragment the bezoar with the use of snares and baskets but only superficial pieces were removed, leaving the bezoar practically intact. Trying to disintegrate the bezoar, we injected, via a variceal needle, inside it 100 ml of hydrogen peroxide (H_2_O_2_) 3% solution. 24 hours later, we repeated the endoscopy and using a modified needle-knife (length of cutting wire 20 mm versus 5 mm of a conventional needle-knife) we performed fragmentation and removal of the bezoar.

## Discussion

The majority of gastric bezoars occur in patients who have undergone previous gastric surgery [2]. Loss of antral and pyloric function because of partial gastric resection and reduced gastric motility following vagotomy are major causes of gastric stasis [1,2]. Other predisposing conditions are impaired mastication, gastroparesis/hypochlorhydria, anatomic abnormalities such as diverticula or gastric outlet obstruction, inadequate fluid intake leading to dehydration and inspissation of enteric feeding formula [1,2].

The clinical presentation of gastric bezoars includes abdominal pain (70%), vomiting and nausea (64%), and early satiety [1-4]. Obstructive symptoms may be intermittent, owing to a ball valve mechanism of obstruction [2]. In some cases the initial presentation may be that of iron deficiency anemia The diagnosis is made by abdominal ultrasound, computed tomography, barium meal examination or endoscopy [5].

Current management includes conservative treatment (meaning waiting for them to disintegrate and pass spontaneously) if the bezoars are small, which however carries the risk of small bowel obstruction in patients who have had gastrectomy; medical treatment with enzymes and prokinetic agents [6]; endoscopic management; and surgical removal. Huge hard bezoars usually require mechanical treatment [3]. Operation is necessary if endoscopic removal fails. Endoscopic management includes enzymatic dissolution by injecting cellulase, use of a water jet, a drill device, tripod forceps, polypectomy snare plus diathermy, Dormia basket, mechanical lithotriptor, or neodymium-yttrium-aluminium-garnet (Nd:YAG) [1-3].

Our case is very intriguing because the consumption of honeycomb has not been reported to lead to gastric bezoar formation. Moreover, the honeycomb-bezoar was very hard to be cut with a snare or basket. We injected inside the bezoar, 100ml H_2_O_2_ 3% solution via a variceal needle. The aim of this injection was the contribution of H_2_O_2_ in disintegration of the bezoar. The endoscopy was repeated 24 hours later. Using a modified needle-knife (bezotome) and monopolar cutting current we were able to incise the bezoar into fragments, which were easily retrieved.

## Conclusion

Our case shows that even a huge solid bezoar with hard consistency does not need to be operated on. By using sophisticated endoscopic techniques the fragmentation and removal of such bezoars is feasible.
